# The New Cardio-Oncology Frontier in Hematologic Cancers: Cardiovascular Toxicities of CAR-T Cells and Bispecific T-Cell Engagers

**DOI:** 10.3390/jcm15166371

**Published:** 2026-08-18

**Authors:** Andrea Tedeschi, Nicolò Pasini, Marco Talassi, Federico Barocelli, Iacopo Fabiani, Vincenzo Quagliariello, Nicola Maurea, Maria Laura Canale, Stefano Oliva, Giampaolo Niccoli, Daniela Aschieri

**Affiliations:** 1 Cardiology Unit, Ospedale Guglielmo da Saliceto, 29121 Piacenza, Italy; d.aschieri@ausl.pc.it; 2 Cardiology Unit, Parma University Hospital, 43126 Parma, Italy; nicolo.pasini@unipr.it (N.P.); marco.talassi@unipr.it (M.T.); federico.barocelli@gmail.com (F.B.); giampaolo.niccoli@unipr.it (G.N.); 3 Cardiology Division, Fondazione Toscana Gabriele Monasterio, 56124 Pisa, Italy; iacopofabiani@gmail.com; 4 Division of Cardiology, Istituto Nazionale Tumori-IRCCS-Fondazione G. Pascale, 80131 Napoli, Italy; quagliariello.enzo@gmail.com (V.Q.); n.maurea@istitutotumori.na.it (N.M.); 5 Cardiology Unit, Versilia Hospital, 55041 Lido di Camaiore, Italy; marialauracanale@icloud.com; 6 Cardiology Unit, Istituto Nazionale Tumori, 70124 Bari, Italy; stefanoliva66@gmail.com

**Keywords:** cardio-oncology, CAR-T cell therapy, bispecific T-cell engagers, cytokine release syndrome, cardiovascular toxicity

## Abstract

Chimeric antigen receptor T-cell therapy and bispecific T-cell engagers have transformed treatment of relapsed and refractory hematologic malignancies, achieving unprecedented response rates. However, their clinical adoption has revealed a complex spectrum of cardiovascular toxicities, differing in frequency and pattern between the two technologies. Manifestations range from common hemodynamic perturbations—hypotension and tachycardia—to severe events, including malignant arrhythmias, left ventricular dysfunction, myocardial infarction, and cardiogenic shock. These complications seem to have different pathophysiological pathways that are yet to be completely understood: on the one hand, they are frequently intertwined with cytokine release syndrome, the hallmark immune complication of T-cell-redirecting therapies, as seen with chimeric antigen receptor T-cell therapy; on the other, a substantial proportion of cardiovascular events—particularly with bispecific T-cell engagers—occur independently of cytokine release syndrome. Proposed cardiotoxic mechanisms include on-target, off-tumor antigen recognition and consequent damage; interleukin-6-driven systemic inflammation; and off-target, off-tumor antigen cross-reactivity. Effective management requires proactive baseline risk stratification, serial cardiac biomarker monitoring, and timely immunosuppressive intervention—primarily tocilizumab—to mitigate cytokine release syndrome-driven injury. Despite rapid clinical expansion, critical gaps remain: long-term cardiovascular outcomes are poorly characterized, validated surveillance protocols are lacking, and cardiovascular endpoints are rarely included in pivotal trials. This narrative review appraises the pathophysiology, clinical spectrum, and management of cardiovascular toxicities associated with these therapies, aiming to define this emerging cardio-oncology frontier, inform multidisciplinary care frameworks and propose a clinical management algorithm.

## 1. Introduction

**Figure 1:**
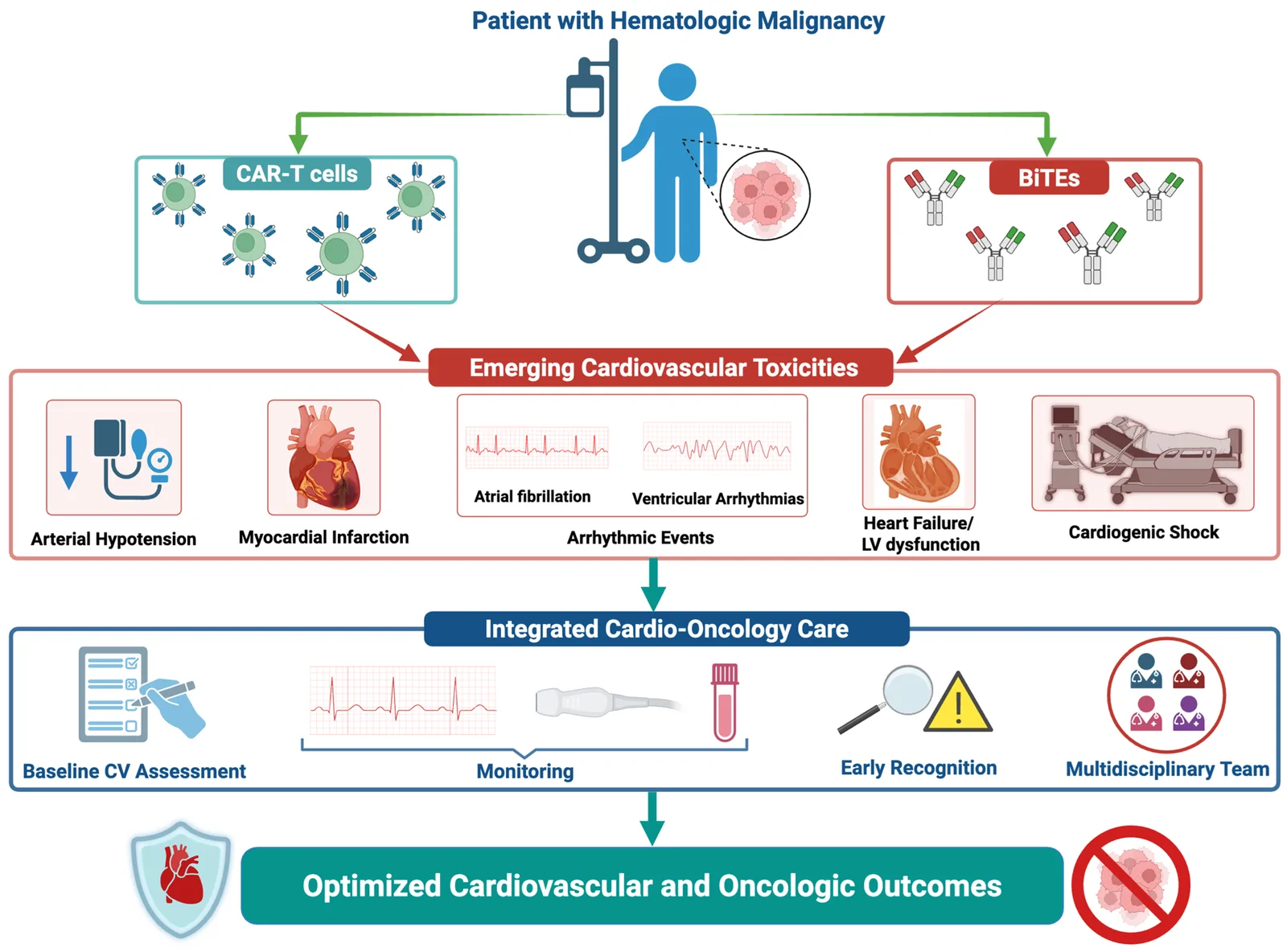
Central Illustration. Integrated cardio-oncology care for CAR-T and BiTE therapies. The figure illustrates the clinical pathway of patients with hematologic malignancies receiving chimeric antigen receptor T-cell (CAR-T) therapies or bispecific T-cell engagers (BiTEs), who may develop emerging cardiovascular toxicities during treatment. Integrated cardio-oncology care, based on baseline cardiovascular assessment, longitudinal monitoring, early recognition of cardiovascular complications, and multidisciplinary management, is essential to identifying and managing treatment-related cardiovascular toxicities. This approach aims to optimize both cardiovascular and oncologic outcomes in patients receiving CAR-T and BiTE therapies. Abbreviations: BiTEs, bispecific T-cell engagers; CAR-T, chimeric antigen receptor T cell; CV, cardiovascular; LV, left ventricular. This figure was created with BioRender.com.

The treatment landscape of hematologic malignancies has been profoundly transformed by the advent of T-cell-redirecting immunotherapies, most notably chimeric antigen receptor (CAR)-T cell therapy and bispecific T-cell engagers (BiTEs). These agents have demonstrated remarkable efficacy in patients with relapsed or refractory disease, achieving response rates previously unattainable with conventional approaches and, in some cases, enabling durable remissions. However, their expanding clinical adoption has uncovered a complex spectrum of cardiovascular (CV) toxicities that demands dedicated attention from the cardio-oncology community [1,2]. Furthermore, many of these patients present with substantial comorbidity burdens and pre-existing CV risk factors, often compounded by prior exposure to cardiotoxic chemotherapeutic regimens, underscoring the clinical complexity of optimizing hematologic outcomes while preserving cardiovascular health in this population. Cardio-oncology has traditionally centered on the CV sequelae of conventional chemotherapeutics—most prominently anthracycline-induced cardiotoxicity [3]—and, more recently, on immune checkpoint inhibitor-associated myocarditis and atherosclerotic disease [4,5]. However, the cardiovascular implications of T-cell-redirecting immunotherapies represent a distinct and rapidly evolving frontier that requires dedicated investigation. CAR-T cell therapy has been associated with a unique profile of cardiovascular complications intimately linked to cytokine release syndrome (CRS) and systemic inflammation. Early clinical observations have documented acute cardiotoxicity events—including arrhythmias, left ventricular dysfunction, and hemodynamic compromise—occurring in the context of the profound immune activation that characterizes CAR-T cell expansion [6]. The CARdio-Tox prospective study further revealed temporal patterns of both systolic and diastolic changes that may evolve dynamically during and after CAR-T cell infusion [7]. Beyond the acute phase, emerging evidence suggests that CAR-T therapy may trigger sustained vascular inflammation in patients treated for hematologic malignancies, raising concerns about accelerated atherosclerosis and long-term CV sequelae extending well beyond the initial treatment window [8,9,10]. As patients achieve durable remissions with these novel agents, the burden of late-onset CV toxicity—including heart failure (HF), coronary artery disease (CAD), and vascular dysfunction—demands systematic longitudinal surveillance. In contrast, the CV toxicity profile of BiTEs remains comparatively undercharacterized. Unlike CAR-T therapy, a substantial proportion of CV adverse events associated with BiTEs appear to occur independently of CRS, suggesting distinct and incompletely understood pathophysiological mechanisms that warrant dedicated investigation. Comprehensive frameworks integrating the cardiotoxic potential of both therapy classes are currently lacking. This narrative review aims to provide a systematic appraisal of the pathophysiology, clinical spectrum, and management of CV toxicities associated with CAR-T cells and BiTEs in hematologic malignancies, synthesizing current evidence and identifying knowledge gaps, with the ultimate goal of informing the development of integrated cardio-oncology frameworks for this emerging therapeutic frontier. Unlike previous cardio-oncology reviews focused on traditional chemotherapy, immune checkpoint inhibitors, or CAR-T therapy alone, this review provides a direct horizontal comparison between CAR-T cells and bispecific T-cell engagers (BiTEs). Additionally, this work bridges mechanistic insights with practical care by incorporating novel risk stratification tools (e.g., the CART-7 score) and proposing a phase-specific clinical algorithm—covering baseline evaluation, acute monitoring, and long-term surveillance. The diagnostic and therapeutic pathway for patients undergoing these treatments is summarized in Figure 1.

## 2. Therapy Landscape

CAR-T cell therapy and BiTEs represent two distinct but mechanistically related approaches to T-cell-redirecting immunotherapy that have transformed the management of hematologic malignancies over the past decade. Both classes exploit the cytotoxic potential of T lymphocytes against tumor-associated antigens but differ substantially in their manufacture, pharmacokinetics, and toxicity profiles.

### 2.1. CAR-T Therapy

CAR-T cell therapy involves the ex vivo genetic engineering of a patient’s own T cells to express a chimeric antigen receptor—a synthetic construct combining an extracellular antigen-binding domain with intracellular signaling components that drive T-cell activation and cytotoxicity upon antigen engagement. The first FDA-approved product, tisagenlecleucel, received approval in 2017 for pediatric and young adult patients with relapsed or refractory B-cell acute lymphoblastic leukemia. Since then, the field has expanded considerably, with multiple approved products covering indications across B-cell lymphomas, multiple myeloma, and acute lymphoblastic leukemia. CAR-T constructs are commonly classified by generation, reflecting progressive refinements in their intracellular signaling architecture. First-generation CAR-T incorporated extracellular domains (antibody-derived single-chain variable fragment) engineered to only recognize and bind directly to the CD19 protein found on the surface of B cells, resulting in limited T-cell proliferation and persistence [11]. Second-generation constructs—which include the majority of currently approved products—integrated a single costimulatory domain (CD28 or 4-1BB), substantially improving T-cell expansion, persistence, and antitumor activity [12]. Third-generation CARs combined multiple costimulatory domains, though without consistent evidence of superior clinical efficacy over second-generation designs [13]. Fourth- and fifth-generation constructs have introduced additional functional enhancements, including the capacity to secrete proinflammatory cytokines or engage innate immune components, broadening antitumor activity and enabling recognition of a wider spectrum of tumor-associated antigens [14]. Target selection represents another key dimension of CAR-T classification. CD19 remains the most extensively studied and clinically utilized target [11], followed by B-cell maturation antigen (BCMA) in multiple myeloma. Additional targets under active investigation include CD20, CD22, and CD138, among others. To overcome limitations such as antigen downregulation and immune evasion, multi-target CAR-T constructs capable of recognizing more than one antigen simultaneously are under development [12]. Complementary strategies include combination with immune checkpoint inhibitors to enhance CAR-T persistence—as demonstrated in patients with malignant pleural disease treated with mesothelin-directed CAR-T and pembrolizumab [15,16]—genetic disruption of inhibitory pathways such as PD-1/PD-L1 via CRISPR/Cas9 editing [17,18], and adaptor CAR-T (AdCAR-T) platforms that employ intermediary molecules to redirect a universal CAR construct toward different tumor antigens, increasing therapeutic flexibility [19].

### 2.2. Bispecific T-Cell Engagers

Unlike CAR-T therapy, BiTEs are recombinant bispecific antibody constructs that simultaneously bind to a tumor-associated antigen on malignant cells and CD3ε on endogenous T lymphocytes, physically bridging the two cell types and inducing polyclonal T-cell activation and tumor cell lysis independent of major histocompatibility complex presentation. A critical practical advantage over CAR-T therapy is that BiTEs are “off-the-shelf” agents that do not require individualized ex vivo T-cell manufacturing, enabling more immediate clinical availability. Blinatumomab, a CD19 × CD3 bispecific construct, was the first BiTE to receive regulatory approval, granted by the FDA in 2014 for relapsed or refractory B-cell acute lymphoblastic leukemia. The class has since expanded rapidly, with multiple agents receiving FDA or EMA approval for hematologic malignancies [20], with indications spanning B-cell lymphomas and multiple myeloma. Approved agents for lymphoma include epcoritamab (CD20 × CD3) and glofitamab (CD20 × CD3), while the multiple myeloma landscape includes teclistamab, elranatamab, and talquetamab, targeting BCMA × CD3, BCMA × CD3, and GPRC5D × CD3 respectively. Mosunetuzumab has received approval for follicular lymphoma. Administration schedules typically involve an initial step-up dosing phase—designed to mitigate cytokine release syndrome—followed by weekly or biweekly maintenance dosing, with extended intervals under investigation in responding patients [20]. Unlike CAR-T therapy, which represents a single intervention with a potentially durable effect, BiTEs require continuous or intermittent administration, with distinct implications for cumulative toxicity and long-term CV surveillance. The primary mechanism of off-tumor toxicity in BiTE therapies relates to antigen expression on normal tissues, which can lead to on-target, off-tumor organ damage [21,22]. Several strategies to improve selectivity are under investigation, including prodrug BiTE constructs selectively activated within the tumor microenvironment and dual tumor-specific binding domain designs [23]. 

## 3. Pathophysiology of Cardiotoxicity

**Figure 2:**
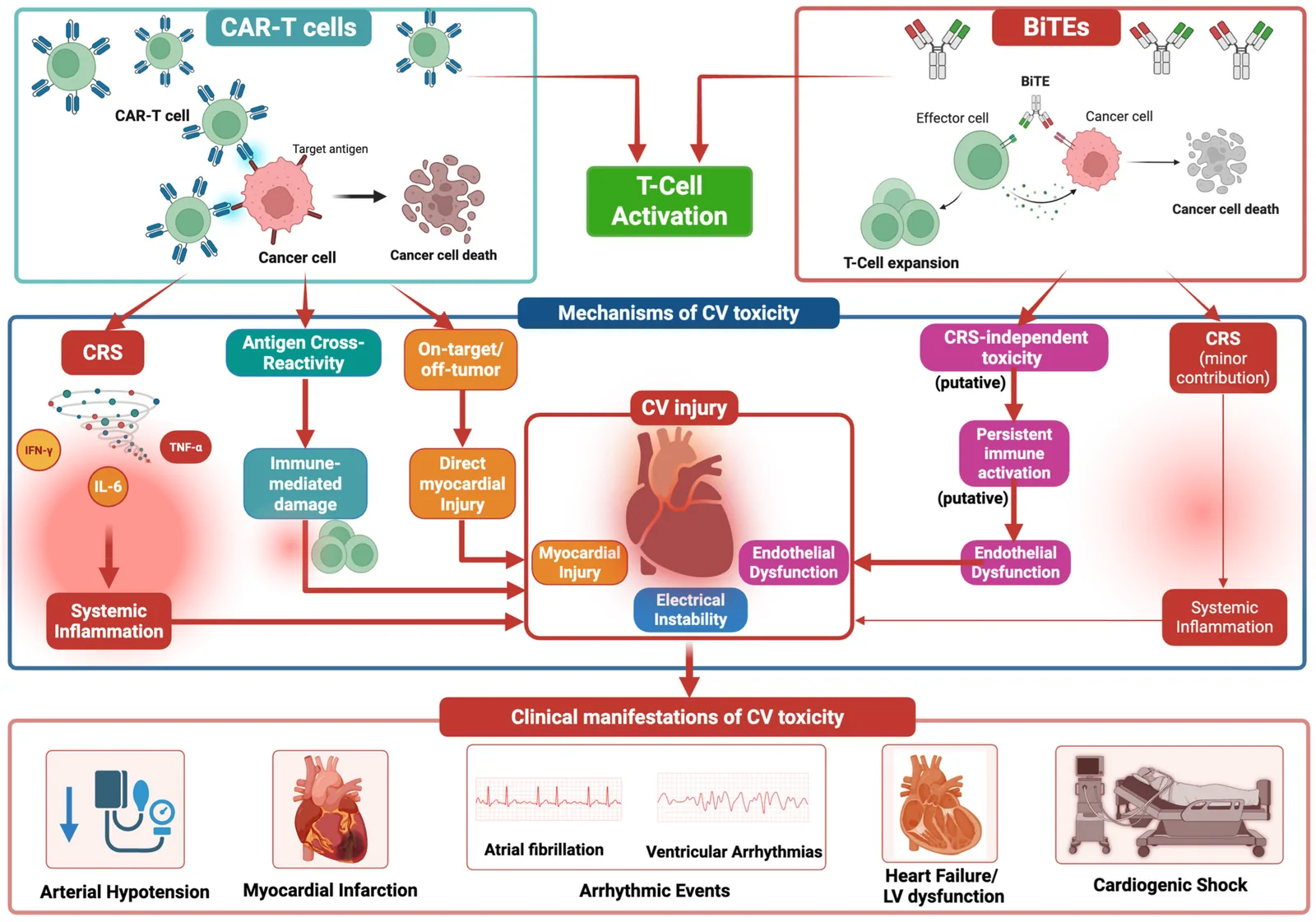
Mechanisms of cardiovascular toxicity associated with chimeric antigen receptor T-cell (CAR-T) therapy and bispecific T-cell engagers (BiTEs). The figure summarizes the proposed mechanisms underlying cardiovascular toxicity associated with T-cell-redirecting immunotherapies. CAR-T-related cardiovascular toxicity is predominantly driven by cytokine release syndrome (CRS), although additional mechanisms such as on-target/off-tumor toxicity and antigen cross-reactivity may contribute to myocardial injury. In contrast, cardiovascular toxicity associated with BiTEs appears to be less dependent on CRS and may involve putative CRS-independent pathways, including persistent immune activation and endothelial dysfunction. These mechanisms ultimately converge on cardiovascular injury, resulting in a broad spectrum of clinical manifestations. In the BiTE pathway, the thinner arrow connecting CRS to cardiovascular injury reflects the currently proposed lesser contribution of CRS to cardiovascular toxicity compared with putative CRS-independent mechanisms. Abbreviations: BiTEs, bispecific T-cell engagers; CAR-T, chimeric antigen receptor T cell; CRS, cytokine release syndrome; CV, cardiovascular; HF, heart failure; IFN-γ, interferon gamma; IL-6, interleukin-6; LV, left ventricular; TNF-α, tumor necrosis factor-alpha. This figure was created with BioRender.com.

CAR-T cell therapy and BiTE therapy have both been associated with CV adverse events, contributing to an increased overall CV risk profile in treated patients. Three principal mechanisms have been proposed to explain CAR-T-related cardiotoxicity: direct myocardial damage, systemic inflammatory responses, and antigen cross-reactivity. The first mechanism involves direct cytotoxic effects mediated by engineered T cells recognizing antigens expressed on myocardial tissue—a phenomenon classified as “on-target, off-tumor” toxicity [24]. This occurs when the intended target antigen, rather than being truly tumor-specific, is also expressed on normal cardiac tissue, rendering cardiomyocytes susceptible to CAR-T-mediated cytotoxicity [11,25,26]. This represents a fundamental challenge in CAR-T therapy because most tumor-associated antigens are not truly tumor-specific but rather are self-antigens expressed at higher levels on malignant cells [11,12]. The CAR-T cells function exactly as designed—they successfully recognize their target antigen—but cause collateral damage to healthy organs that share expression of that antigen [13,26]. It should be noted, however, that clinically overt on-target, off-tumor cardiac toxicity with currently approved CD19- and BCMA-directed CAR-T products is extremely rare, as these antigens are not meaningfully expressed on cardiac tissue. This mechanism is therefore primarily a theoretical concern for next-generation constructs targeting novel antigens with broader tissue expression profiles. The second—and clinically most relevant—mechanism is cytokine release syndrome (CRS), a systemic inflammatory response resulting from the interaction between CAR-T cells and tumor cells within the tumor microenvironment, classified as an “on-target, on-tumor” adverse effect [27]. This interaction triggers a cascade of proinflammatory mediators, including interleukin-6 (IL-6), tumor necrosis factor-alpha (TNF-α), and nitric oxide, driving systemic inflammation and immune activation [14]. Among these, IL-6 plays a central and well-characterized role in CRS-associated CV injury. Clinically, CRS typically manifests 2–3 days post-infusion—though onset may occur within hours or be delayed up to 10–15 days—and presents with fever, hypotension, tachycardia, and hypoxia. The overall incidence of CRS ranges from 35% to 93%, with severe cases occurring in 1–47% of CAR-T recipients [12,15]. Serious CV events associated with CRS include atrial fibrillation (AF), ventricular tachycardia, cardiac arrest, HF, and capillary leak syndrome [12,15,16,17]. Approximately 10–15% of patients may develop cardiomyopathy in the context of high-grade CRS, and CV events occur in up to 20% of patients with high-grade CRS [18]. Both the American Society for Transplantation and Cellular Therapy and the Common Terminology Criteria for Adverse Events have established grading systems for CRS severity, which are essential for guiding therapeutic management [17]. CRS may also involve the central nervous system, resulting in a condition known as immune effector cell-associated neurotoxicity syndrome (ICANS), which typically occurs 4–10 days post-infusion and is characterized by heterogeneous neurologic symptoms, including encephalopathy, delirium, aphasia, tremor, motor dysfunction, and, in severe cases, seizures and cerebral edema [1,12]. Recognition of ICANS is critical, as it carries distinct therapeutic and prognostic implications compared with systemic CRS alone. While IL-6 is central to CRS, myocardial and endothelial injury during CRS results from a synergistic cytokine network involving interleukin 1 beta (IL-1β), TNF-α, interferon gamma (IFN-γ), and interleukin 8 (IL-8). TNF-α and IFN-γ act synergistically with IL-6 trans-signaling to disrupt endothelial barrier integrity and upregulate cell adhesion molecules [19,20]. Local IL-8 production recruits and activates neutrophils, while TNF-α and IL-1β downregulate endothelial nitric oxide synthase and induce endothelial apoptosis [21]. Together, these pathways drive systemic capillary leak, microvascular thrombosis, and refractory hypotension. TNF-α and IL-1β directly blunt cardiomyocyte β-adrenergic responsiveness, disrupting intracellular calcium handling and causing acute contractile dysfunction [20,21,22]. Simultaneously, IFN-γ amplifies inflammatory signaling, promotes mitochondrial reactive oxygen species production, and triggers opening of the mitochondrial permeability transition pore [23]. The third proposed mechanism may be attributed to the lack of selectivity, especially with first-generation CAR-T. It is an “off-target, off-tumor” effect in which CAR-T cells recognize antigens that were not intended as targets—antigens that are expressed on normal tissues but not on tumor cells, including cardiac muscle [28,29,30,31]. This can arise through several distinct pathways: molecular mimicry, whereby the CAR or T-cell receptor recognizes structurally similar epitopes on unrelated proteins; antigen-independent activation through interactions between CAR constructs and innate immune cells; and tonic signaling, in which constitutive CAR activation occurs in the absence of antigen engagement [30,31,32]. The risk is particularly high when using affinity-enhanced or modified T-cell receptors, which may have broader peptide recognition than naturally occurring receptors [12,18]. This kind of cross-reactivity and its potentially dangerous effects are well documented in the current literature and demonstrated using different genetic technologies. One example could be the cross-reaction of a structurally similar peptide derived from titin, a sarcomeric protein expressed in cardiac muscle, with affinity-enhanced T-cell receptors originally designed to target MAGE-A3, causing fatal cardiogenic shock [28]. While such direct cardiotoxicity remains rare with current CD19- and BCMA-directed CAR-T products, it underscores the potential for off-target effects when targeting antigens with tissue expression beyond the tumor [33]. While this mechanism has been less commonly implicated in current commercial CAR-T products, it remains a theoretical concern as CAR-T therapy expands to target novel antigens and solid tumors. In contrast, the cardiotoxic mechanisms of BiTE therapy are less well characterized and appear to differ in important aspects from those of CAR-T. While CRS remains a recognized driver of CV injury with BiTEs, a particularly notable finding from pharmacovigilance data is that the majority of CV adverse events associated with BiTE therapy occur independently of CRS [34], suggesting that distinct and incompletely understood pathophysiological mechanisms are involved beyond cytokine-mediated injury. Whether these reflect direct endothelial or myocardial effects of sustained T-cell activation, immune complex deposition, or other mechanisms remains to be defined. The full characterization of BiTE-associated cardiotoxicity—including its incidence, clinical spectrum, and temporal patterns—is addressed in detail in the following sections. The mechanisms of cardiotoxicity are illustrated in Figure 2.

## 4. Clinical Spectrum

CAR-T and BiTE therapies are associated with a broad and heterogeneous spectrum of CV adverse events (CVAEs), although the currently available evidence remains partly inconsistent because of the predominance of retrospective studies, heterogeneous patient populations, and variability across meta-analyses. These immune-based therapies may induce acute hemodynamic instability, rhythm disturbances [27], myocardial injury, ischemic and thrombotic complications, pericardial involvement, and other CV toxicities, frequently in association with CRS.

### 4.1. Pivotal Studies of CAR-T and BiTE Therapies 

The pivotal clinical trials investigating CAR-T and BiTE therapies primarily reported CV toxicities in the context of CRS, with hypotension and tachycardia representing the most frequently observed manifestations. In the ZUMA-1 trial evaluating axicabtagene ciloleucel (axi-cel), the principal CV adverse events were hypotension and tachycardia [35]. The study enrolled 111 patients, including 77 with diffuse large B-cell lymphoma and 24 with primary mediastinal B-cell lymphoma or transformed follicular lymphoma. Among the 111 patients who received axi-cel, all experienced adverse events, which were grade 3 or higher in 95% of cases. CRS occurred in 93% of patients, with grade ≥ 3 CRS reported in 13% (9% grade 3, 3% grade 4, and 1% grade 5). Hypotension was observed in 59% of patients with CRS, while vasopressor support was required in 17% of all treated patients [35]. Similarly, the JULIET trial investigating tisagenlecleucel in relapsed or refractory diffuse large B-cell lymphoma reported a substantial incidence of CRS-related hemodynamic complications [36]. A total of 93 patients received the infusion, and 22% developed grade 3–4 CRS requiring intensive supportive measures, including oxygen supplementation (24%), endotracheal intubation (7%), high-dose vasopressors (6%), and dialysis (5%). Hypotension of any grade occurred in 26% of patients, whereas tachycardia was reported in 11% [36]. Comparable findings emerged from the Phase 2 KarMMa study evaluating idecabtagene vicleucel in relapsed or refractory multiple myeloma [37]. Hemodynamic adverse events were again frequently observed, with hypotension occurring in 16% of patients. In addition, electrolyte abnormalities potentially predisposing to arrhythmias were commonly reported, including hypokalemia (35%), hypophosphatemia (30%), hypocalcemia (27%), and hypomagnesemia (23%). However, specific data regarding arrhythmic events were not reported [37]. Clinical trials investigating BiTE therapies have similarly described hypotension and tachycardia as the most common CV adverse events. In the Phase 3 TOWER trial evaluating blinatumomab versus chemotherapy in advanced acute lymphoblastic leukemia [38], 267 patients received blinatumomab. Hypotension occurred in 12% of patients and tachycardia in 6.7%, while isolated cases of AF and atrial flutter were also documented. Consistent findings with similar incidences were reported in the Phase 2 BLAST study on blinatumomab [39]. In contrast, the Phase 1–2 study evaluating teclistamab in relapsed or refractory multiple myeloma suggested a lower incidence of clinically significant hemodynamic toxicity [40]. Although 72.1% of the 165 treated patients developed CRS-related symptoms, vasopressor support was required in only one patient, indicating a comparatively limited CV impact in terms of severe hypotensive events [40]. As reported before, these mechanisms and their real pathophysiology, as well as their incidence, remain uncertain, and prospective studies are needed.

### 4.2. Cardiovascular Adverse Events in CAR-T and BiTE Therapies: Evidence from Dedicated Studies

Beyond pivotal trials, dedicated observational studies and meta-analyses have provided a more granular characterization of the CV toxicity profiles of both therapy classes. In a comprehensive review, Marar et al. [41] described the main cardiovascular manifestations, including tachycardia, troponin elevation, reduction in left ventricular ejection fraction (LVEF), decompensated HF with pulmonary edema, new-onset arrhythmias, and severe hypotension progressing to shock. More recent quantitative data from the meta-analysis by Maleki et al. [42] reported high incidences of CVAEs, largely associated with CRS of grade ≥ 2. Importantly, patients experiencing CVAEs showed a greater decline in LVEF, reinforcing the need for structured CV monitoring during CAR-T therapy [42]. However, real-world data appear to suggest lower event rates: in a retrospective study, Lefebvre et al. [43] reported a 21% incidence of major adverse CV events (MACEs) at 12 months, mainly driven by symptomatic HF. Despite discrepancies in incidence, studies consistently describe a relatively early onset of CVAEs, typically within 5–21 days after infusion [41,43,44,45]. Furthermore, delayed treatment of CRS appears to correlate with an increased risk of CV complications [41]. BiTE therapy also demonstrates a significant, though somewhat less extensively characterized, CV toxicity profile. Data derive mainly from a few dedicated studies and from the US-FDA reporting system, making them subject to important possible biases—such as duplicate reports or underreporting—and making it difficult to establish real-world prevalence. An analysis of data from the US Food and Drug Administration’s Adverse Event Reporting System (FAERS) by Sayed et al. [45] showed that 20.4% of 3668 reported adverse events were cardiovascular in nature. The most frequently reported events included bleeding, thromboembolic complications, hypotension, shock, and HF, with additional manifestations such as atrial fibrillation/flutter, pericardial effusion, myocardial infarction, ventricular tachyarrhythmias, myocarditis, pericarditis, and rare cases of sudden death [45]. Similarly to CAR-T therapies, CVAEs associated with BiTE treatment tend to occur early after administration, within a median timeframe of 5–21 days [41,43,44,45]. In the recent study by Itzhaki Ben Zadok O et al. [44], the overall incidence of CVAEs was 10.4%, including left ventricular dysfunction, new-onset AF, ACS, AF with rapid ventricular response, HF with preserved ejection fraction, supraventricular tachycardia, cerebrovascular events, ventricular arrhythmias, and high-degree atrioventricular block, with the most common being new left ventricular dysfunction (2.3%) and new-onset AF (2.1%) [44]. Overall, although variability in reported incidence persists, available data allow the delineation of a consistent clinical spectrum of cardiotoxicities for both CAR-T and BiTE therapies. Two prospective ongoing studies aim to present more objective and real-world data on cardiotoxicity for products based on CAR-T and assess their cardiac safety (NCT06106776 and NCT05130489).

## 5. Quantitative Evidence and Prognostic Impact of Cardiovascular Toxicity

While the preceding section characterized the clinical spectrum of CV toxicities as reported across pivotal and observational studies, a distinct body of evidence—derived from dedicated registries, pharmacovigilance databases, and quantitative meta-analyses—has sought to define the precise incidence, signal strength, and prognostic weight of CV adverse events associated with CAR-T cell therapy and BiTEs. Notably, CV adverse events have been reported in approximately 20% of patients receiving either therapy class, though the spectrum, severity, and prognostic implications differ in important ways between the two. Even though these findings derive from different sources, reducing the possibility of extrapolating definite conclusions, they all go in similar directions. The key findings from these studies are summarized below and in Table 1 and Table 2.

### 5.1. CAR-T Therapy

Incidence rates of CV toxicity in CAR-T therapy vary significantly between clinical trials and real-world data. In a meta-analysis of 13 studies, the most observed cardiac events were left ventricular dysfunction (8.68%; 95% CI 2.26–17.97%) and supraventricular arrhythmia (7.79%; 95% CI 4.87–11.27%). Serious adverse cardiac events were less common: ventricular arrhythmia (0.66%), myocardial infarction (0.62%), and CV death (0.63%) [46]. Major adverse cardiac events have been reported in up to 16% of patients following CAR-T, with the majority being arrhythmias, though myocardial infarction and acute HF also occur [15]. A single-center registry of 137 patients found that 12% experienced CV events (six CV deaths, six decompensated HF, five arrhythmias), with all events occurring in patients with grade ≥ 2 CRS and 95% occurring after elevated troponin [47]. Among patients with high-grade CRS, 28% experienced decreased ejection fraction [47]. A meta-analysis comparing Phase 1/2 to Phase 3 trials revealed significant underreporting of CV adverse events in later-phase studies. Phase 1/2 trials reported an all-grade CV adverse event rate of 21.83% and a grade ≥ 3 rate of 8.82%, while Phase 3 trials showed only 3.4% grade ≥ 3 CV events in CAR-T groups versus 2.1% in control groups (OR 1.44, *p* = 0.29) [48], a discrepancy likely reflecting differences in patient selection, monitoring intensity, and endpoint adjudication rather than true differences in toxicity. Post-marketing surveillance using FAERS identified 2657 CAR-T reports, including 546 cardiovascular and pulmonary adverse events (20.5%). Cardiovascular and Pulmonary Adverse Events (CPAEs) overlapped with CRS in 68.3% of reports, though 31.7% occurred independently of CRS [16]. CAR-T was associated with significant overreporting of tachyarrhythmias (n = 74 [2.8%], Adjusted Reporting Odds Ratio [adj.ROR] = 2.78 [95% CI 2.21–3.51]), cardiomyopathy (n = 69 [2.6%], adj.ROR = 3.51 [2.42–5.09]), pleural disorders (n = 46 [1.7%], adj.ROR = 3.91 [2.92–5.23]), and pericardial diseases (n = 11 [0.4%], adj.ROR = 2.26 [1.25–4.09]) [16]. The fatality rate of cardiovascular and pulmonary adverse events was 30.9%, with rates ranging from 27% to 41% for specific events [16]. Finally, a multicenter registry found that severe CV events (HF, cardiogenic shock, or myocardial infarction) were independently associated with increased overall mortality (HR 2.8, 95% CI 1.6–4.7) and non-relapse mortality (HR 3.5, 95% CI 1.4–8.8) [49]. A summary of the previously cited studies is shown in Table 1.

**Table 1:** Summary of studies involving CAR-T therapies.

Authors, Year (Study)	Study Design	Study Population	Main CV Findings (Adverse Events & Frequencies)
CAR-T Cell Therapy—Pivotal & Observational Studies			
Neelapu et al., 2017 [35] (ZUMA-1) CAR-T	Phase 2, single-arm RCT	111 patients with refractory large B-cell lymphoma (DLBCL, PMBCL, TFL) Treatment: axicabtagene ciloleucel (anti-CD19 CAR-T)	• CRS: 93% (grade ≥ 3: 13%; grade 3: 9%, grade 4: 3%, grade 5: 1%) • Hypotension: 59%, 17% requiring vasopressor support • Tachycardia: 39% of patients • All patients experienced ≥ 1 adverse event; grade ≥ 3 in 95%
Schuster et al., 2019 [36] (JULIET) CAR-T	Phase 2, single-arm trial	93 patients with relapsed/refractory DLBCL Treatment: tisagenlecleucel (anti-CD19 CAR-T)	• Grade 3–4 CRS: 22% • Hypotension (any grade): 26% • Tachycardia: 11% • O_2_ supplementation: 24%; endotracheal intubation: 7%; high-dose vasopressors: 6%; dialysis: 5%
Munshi et al., 2021 [37] (KarMMa) CAR-T	Phase 2, single-arm trial	128 patients with relapsed/refractory multiple myeloma Treatment: idecabtagene vicleucel (ide-cel, anti-BCMA CAR-T)	•CRS: 84% of patients (grade 1: 48%, grade 2: 30%, grade ≥ 3: 5%) • Hypotension: 16% • Hypokalemia: 35%
Alvi et al., 2019 [47] CAR-T	Single-center retrospective registry	137 adults treated with CAR-T for hematologic malignancies	• CV events: 12% (6 CV deaths, 6 decompensated HF, 5 arrhythmias) • All CV events in patients with grade ≥ 2 CRS • 95% of CV events preceded by elevated troponin • Decreased EF in 28% of high-grade CRS patients • Each 12 h delay to tocilizumab → 1.7-fold increase in CV event risk
Lefebvre et al., 2020 [43] CAR-T	Retrospective single-center cohort	145 adult patients receiving anti-CD19 CAR-T cell therapy	• MACEs: 21% (mainly symptomatic heart failure) • Median time to MACE was 11 days after CAR-T cell infusion
Goldman et al., 2021 [16] (FAERS analysis) CAR-T	Pharmacovigilance study (FDA FAERS database)	2657 CAR-T reports in FDA Adverse Event Reporting System	• Cardiovascular & pulmonary AEs: 20.5% of reports (n = 546) • 68.3% overlapped with CRS; 31.7% occurred independent of CRS • Tachyarrhythmias: 2.8% (adj.ROR 2.78, 95% CI 2.21–3.51) • Cardiomyopathy: 2.6% (adj.ROR 3.51, 95% CI 2.42–5.09) • Pericardial diseases: 0.4% (adj.ROR 2.26, 95% CI 1.25–4.09) • Fatality rate of CVAEs: 30.9% (range 27–41% for specific events)
Mahmood et al., 2023 [49] CAR-T	Multicenter retrospective registry	202 CAR-T patients; cardiovascular outcomes and biomarkers assessed	• Severe CV events (HF, cardiogenic shock, MI) independently associated with: – Overall mortality: HR 2.8 (95% CI 1.6–4.7) – Non-relapse mortality: HR 3.5 (95% CI 1.4–8.8)
Maleki et al., 2024 [42] (Meta-analysis) CAR-T	Systematic review and meta-analysis	CAR-T recipients across multiple studies	• Arrhythmias: 54% • HF: 30% • Cardiomyopathy: 20% • ACS: 10% • Cardiac arrest: 7% • Events largely associated with CRS grade ≥ 2 • Greater LVEF decline in patients with CV events
Koeckerling et al., 2024 [46] (Meta-analysis) CAR-T	Meta-analysis (13 studies)	1528 patients with advanced hematologic malignancies treated with CAR-T	• LV dysfunction: 8.68% (95% CI 2.26–17.97%) • Supraventricular arrhythmia: 7.79% (95% CI 4.87–11.27%) • Ventricular arrhythmia: 0.66% • MI: 0.62% • CV death: 0.63% • MACEs reported in up to 16% of patients
Myint et al., 2024 [48] (Meta-analysis) CAR-T	Meta-analysis of Phase 1/2 and Phase 3 controlled and single-arm trials	Cancer patients treated with CAR-T cell therapy	• Phase 1/2 trials: all-grade CVAEs 21.83%; grade ≥ 3 CVAEs 8.82% • Phase 3 trials: grade ≥ 3 CVAEs 3.4% (CAR-T) vs. 2.1% (control) [OR 1.44, *p* = 0.29] → Significant underreporting of CVAEs in later-phase studies

Abbreviations: AE, adverse event; adj.ROR, Adjusted Reporting Odds Ratio; BCMA, B-cell maturation antigen; CAR-T, chimeric antigen receptor T-cell; CI, Confidence Interval; CRS, cytokine release syndrome; CV, cardiovascular; CVAEs, Cardiovascular Adverse Events; DLBCL, diffuse large B-cell lymphoma; EF, ejection fraction; FAERS, FDA Adverse Event Reporting System; FDA, Food and Drug Administration; HF, heart failure; HR, Hazard Ratio; LV, left ventricular; MACEs, Major Adverse Cardiovascular Events; MI, myocardial infarction; OR, Odds Ratio; PMBCL, Primary Mediastinal Large B-cell Lymphoma; RCT, Randomized Controlled Trial; TFL, transformed follicular lymphoma.

**Table 2:** Summary of studies involving BiTE therapies.

Authors, Year (Study)	Study Design	Study Population	Main CV Findings (Adverse Events & Frequencies)
Bispecific T-Cell Engagers (BiTEs)—Pivotal & Observational Studies			
Kantarjian et al., 2017 [38] (TOWER) BiTEs	Phase 3 RCT (blinatumomab vs. chemotherapy)	405 patients with advanced acute lymphoblastic leukemia Treatment: blinatumomab	• Hypotension: 12% • Tachycardia: 6.7% • Isolated cases of AF and atrial flutter
Gökbuget et al., 2018 [39] (BLAST) BiTEs	Phase 2, single-arm study	116 adults with B-cell precursor ALL and MRD Treatment: blinatumomab	• Hypotension: 12.1% • Tachycardia: 4.3%
Moreau et al., 2022 [40] (MajesTEC-1) BiTEs	Phase 1–2 study	165 patients with relapsed/refractory multiple myeloma Treatment: teclistamab	• CRS: 72.1% (predominantly grade 1–2) • Hypotension 18% • Low incidence of severe hemodynamic CV toxicity • Cardiac arrhythmia 16%
Itzhaki Ben Zadok et al., 2026 [44] BiTEs	Dual-center retrospective cohort	567 patients treated with bispecific T-cell engagers for hematologic malignancies	• Cumulative CV event incidence: 10.4% (95% CI 8.1–13.1%) • New LV dysfunction: 2.3%; new-onset AF: 2.1% • CV mortality: 0.4% • Only independent baseline predictor: CAD • Grade ≥ 2 CRS/ICANS → significant time-dependent increase in CV event risk • CV events independently associated with higher all-cause mortality
Sayed et al., 2024 [45] (FAERS analysis) BiTEs	Pharmacovigilance study (FDA FAERS database)	3668 BiTE-related adverse event reports	• CVAEs: 20.4% of all reports • 83.3% of CVAEs did not overlap with CRS • Fatal CVAE class signal: adj.ROR 1.29 (95% CI 1.12–1.50) • Teclistamab: fatal CVAE adj.ROR 2.44 (1.65–3.60); myocarditis adj.ROR 25.70 (9.54–69.23); shock adj.ROR 3.63 (2.30–5.74) • Blinatumomab: DIC adj.ROR 3.02 (1.98–4.60); hypotension adj.ROR 1.59 (1.25–2.03)
Tai et al., 2025 [50] (Meta-analysis) BiTEs	Meta-analysis (37 RCTs)	3758 patients treated with bispecific antibodies for hematologic malignancies	• Tachycardia: 10.2% (95% CI 7.6–13.5%; I^2^ = 64.9%) • Cardiac arrhythmias: 12.6% (95% CI 7.8–19.7%; I^2^ = 50.1%) • Hypotension: 13.3% (95% CI 10–17.3%; I^2^ = 76.9%)
Shan et al., 2025 [51] (FAERS analysis) BiTEs	Pharmacovigilance study (FDA FAERS database)	Multiple myeloma patients receiving BiTEs	• CVAEs: 8.4% of all AEs • CV events associated with death in 34% of cases • 60.9% of events occurred within first month • Risk factors: older age, male sex, weight loss, CRS, respiratory failure

Abbreviations: AE, adverse event; AF, atrial fibrillation; ALL, acute lymphoblastic leukemia; adj.ROR, Adjusted Reporting Odds Ratio; BiTEs, bispecific T-cell engagers; CAD, coronary artery disease; CI, Confidence Interval; CRS, cytokine release syndrome; CV, cardiovascular; CVAEs, Cardiovascular Adverse Events; DIC, disseminated intravascular coagulation; FAERS, FDA Adverse Event Reporting System; FDA, Food and Drug Administration; ICANS, immune effector cell-associated neurotoxicity syndrome; LV, left ventricular; MRD, Minimal Residual Disease; RCT, Randomized Controlled Trial.

### 5.2. Bispecific T-Cell Engagers

From 3668 BiTE-related cases reported to FAERS, 747 (20.4%) involved CV adverse events. BiTEs as a class were associated with fatal CV events (adj.ROR 1.29, 95% CI 1.12–1.50) [45]. The association was mainly driven by teclistamab (adj.ROR 2.44 [95% CI 1.65–3.60]). Teclistamab was also associated with a disproportionate risk of myocarditis (adj.ROR 25.70 [95% CI 9.54–69.23]) and shock (adj.ROR 3.63 [95% CI 2.30–5.74]), whereas blinatumomab was associated with a disproportionate risk of disseminated intravascular coagulation (adj.ROR 3.02 [95% CI 1.98–4.60]) and hypotension (adj.ROR 1.59 [95% CI 1.25–2.03]). Consistent with findings from the pathophysiology section, 83.3% of CV adverse events did not overlap with CRS, reinforcing the existence of distinct cytokine-independent mechanisms of cardiotoxicity [34]. In a meta-analysis that included 37 randomized trials, involving a total of 3758 patients, the significant incidence rates were 10.2% for tachycardia (95% CI 7.6–13.5%, *p* = 0.05, I^2^ = 64.9%), 12.6% for cardiac arrhythmias (95% CI 7.8–19.7%, *p* = 0.05, I^2^ = 50.1%), and 13.3% for hypotension (95% CI 10–17.3%, *p* = 0.05, I^2^ = 76.9%) [50]. Notably, in multiple myeloma patients receiving BiTEs, 8.4% of adverse events were cardiovascular, with arrhythmias, myocarditis/pericarditis, cardiomyopathy, shock, and cardiac failure showing positive signal strengths [51]. The majority of CV events occurred within the first month (60.9%) with early-failure type characteristics. CV events were associated with death in 34% of cases. Risk factors included older age, male sex, weight loss, CRS, and respiratory failure [51]. In a dual-center cohort of 567 patients treated with BiTEs, the cumulative incidence of on-treatment CV events was 10.4% (95% CI 8.1–13.1), with left ventricular dysfunction and AF representing the most common presentations [44]. CV mortality was rare (0.4%). CAD was the only baseline variable independently associated with CV events, while development of grade ≥ 2 CRS and/or ICANS was associated with a significant time-dependent increase in CV event risk. Importantly, CV events were significantly associated with higher all-cause mortality, independent of baseline clinical factors and BiTE agent [44]. Long-term cardiovascular effects beyond one year remain poorly characterized for both therapy classes [33]. A summary of CV toxicities in the cited studies is shown in Table 2. Taken together, the evidence discussed above highlights that, despite some overlapping clinical manifestations, CAR-T- and BiTE-associated CV toxicity differ meaningfully in their relationship with CRS, risk factors, and the strength of supporting evidence. Table 3 summarizes and compares these main pathophysiological, clinical, and management differences between the two therapy classes.

## 6. Management (From Risk Stratification to Long-Term Follow-Up)

To date, no international guidelines describe specific recommendations for the management of patients receiving CAR-T or BiTE therapy. Current clinical practice is mainly driven by references to expert consensus, best clinical practice used in the majority of centers and recommendations for other oncology therapies with an impact on cardiovascular disease. The cardiovascular management of patients receiving CAR-T cell therapy or BiTE therapy could be best conceptualized as a continuum spanning three distinct phases: a pre-treatment phase focused on baseline risk stratification and cardiovascular optimization, an intra-treatment phase centered on hemodynamic monitoring and prompt management of CRS-related complications, and a post-treatment phase dedicated to surveillance for late-onset cardiovascular toxicity and long-term follow-up. Each phase carries specific clinical objectives and requires a proactive, multidisciplinary cardio-oncology approach to minimize preventable cardiovascular morbidity without compromising oncologic outcomes.

### 6.1. Baseline Clinical Assessment

Comprehensive CV evaluation prior to treatment with CAR-T or BiTE therapies should include detailed CV history and physical examination, estimation of exercise tolerance, screening for CV disease risk factors (hypertension, diabetes, hyperlipidemia, obesity, smoking), and baseline 12-lead ECG [12]. Cardiac evaluation indications before therapy are likely to vary between institutions; however, assessment often includes exclusion of coronary ischemia and structural heart disease. Patients with pre-existing CVD, multiple cardiovascular (CV) risk factors or active CV symptoms will likely benefit from further risk stratification and optimization of CV status. Echocardiography is the cornerstone of baseline imaging. While not mandated, an echocardiogram prior to CAR-T or BiTE therapy is often performed to assess biventricular systolic function and exclude significant valvular disease [12]. In those with pre-existing CAD or multiple CV risk factors, exercise tolerance should be assessed, and for patients with poor exercise capacity or exertional symptoms, it is reasonable to consider an imaging stress test to rule out occult obstructive CAD [12,52,53]. Patients with obstructive CAD or significant aortic stenosis may be at increased risk of major CV events such as myocardial infarction, ventricular arrhythmia, HF, cardiogenic shock, and even death in the setting of CRS-induced hypotension. Cardiac magnetic resonance may be considered as needed, particularly in patients with inconclusive echocardiographic findings or when more detailed tissue characterization is required [12,52]. Cardiac biomarkers, including troponin and B-type Natriuretic Peptide/N-terminal pro-B-type Natriuretic Peptide (BNP, NT-proBNP), could be useful tools for monitoring during therapy, particularly when triggered by clinical signs and symptoms of high-grade CRS [12]. Patients who experienced severe CV events had higher peak IL-6, C-reactive protein (CRP), ferritin, and troponin levels [49]. Troponin elevation is associated with subsequent CV events, and in one registry, all CV events occurred in patients with grade ≥ 2 CRS, and 95% occurred after elevation of troponin [47]. CRP and ferritin should be measured at baseline (prior to lymphodepleting chemotherapy) to allow for calculation of the CAR-HEMATOTOX score to predict the risk of immune effector cell-associated hematotoxicity (ICAHT) and infection [54]. The CART-7 score is a novel risk-assessment tool derived from a meta-analysis of 1354 patients, designed to predict CV adverse events following CAR-T cell therapy [55]. The scoring system evaluates seven baseline factors—HF, AF, CAD, diabetes, hyperlipidemia, hypertension, and smoking—weighting them based on their relative risk to assign a total score between 0 and 33. Patients are subsequently stratified into low (0–8), moderate (9–17), high (18–25), or very high (26–33) risk categories [55]. However, while promising for clinical planning, the tool currently requires more extensive validation before it can be formally integrated into standard medical practice. To optimize cardiovascular health prior to therapy, clinicians should employ a multifaceted strategy that includes adjusting medications—specifically tapering antihypertensives to mitigate the risk of CRS-induced hypotension—and maintaining low-dose aspirin in patients with an indication for antiplatelet therapy for CAD or in patients with previous percutaneous coronary intervention, until platelet counts drop below 30,000 [12]. Furthermore, patients with pre-existing HF require rigorous volume status monitoring and tailored management plans [34], while those with a significant CV burden should be managed through a multidisciplinary approach that involves the patient in weighing the therapeutic benefits against the risks relative to their hematologic and cardiac prognosis [12].

### 6.2. Management During Infusion

Managing CAR-T cell and BiTE therapies requires vigilant blood pressure monitoring to identify and treat hypotension, which often involves administering intravenous fluids while carefully watching for signs of vascular leak or pulmonary congestion [12]. It is critical to differentiate between various causes of low blood pressure, such as pulmonary embolism, primary cardiac events, or septic shock—the latter of which requires empiric antibiotics, as infection frequently mimics or overlaps with CRS. Prompt intervention for CRS depends on early detection and a structured monitoring approach. For patients exhibiting moderate symptoms, antipyretics are indicated for temperatures of 38.0 °C or higher, while intravenous fluids should be administered to address fever or clinical markers of hypovolemia, such as dizziness, fatigue, or sinus tachycardia. To refine the diagnosis of fluid status, clinicians can utilize ultrasound to measure the diameter of the inferior vena cava. Additionally, it is essential to simultaneously evaluate and treat any potential systemic infections, as their clinical presentation often mimics that of CRS [56]. When CRS becomes severe, patients may require immediate escalation to intensive care for advanced interventions like mechanical ventilation, invasive blood pressure monitoring, and mechanical circulatory support. Furthermore, while CRS-related shock is typically vasodilatory, clinicians should consider mixed or purely cardiogenic shock in patients who do not respond to standard resuscitation; in these complex cases, continuous invasive hemodynamic monitoring is often beneficial to precisely calibrate fluid and vasopressor needs [12]. In cases where hypotension persists despite aggressive fluid resuscitation, the management strategy must expand to include vasopressor therapy, the correction of coagulopathies, and the comprehensive treatment of any developing multi-organ dysfunction [56]. As an FDA-approved first-line treatment for toxicities related to CAR-T therapy, but also for BiTE toxicities, tocilizumab is a monoclonal antibody that mitigates CRS by competitively inhibiting IL-6 from binding to its receptors. By targeting both membrane-bound and soluble IL-6 receptors, the drug effectively blocks classic and trans-signaling pathways, thereby halting the transduction of IL-6 into inflammatory mediators [12]. Institutional practices for the timing of tocilizumab administration vary significantly, as there is currently no universal clinical consensus on exactly which stage of CRS severity should trigger its initiation. Some institutions reserve the drug for severe complications—such as hypotension requiring more than 24 h of pressor support, unstable arrhythmias, elevated troponin levels, or new-onset cardiomyopathy with an LVEF below 40%—while others have shifted toward a more proactive approach. Emerging evidence suggests that early administration at the very first signs of CRS may be safe, leading some practitioners to integrate earlier intervention into their standard clinical workflows [57]. In one study, each 12 h delay in tocilizumab administration increased CV event risk 1.7-fold [47]. While corticosteroids are effective for managing CRS, they are typically utilized as a second-line treatment reserved for cases refractory to tocilizumab due to theoretical concerns that they might impair the antitumor activity of engineered T cells. However, despite these fears regarding the potential for steroids or tocilizumab to decrease CAR-T cell efficacy, data from initial clinical trials have not demonstrated a link between the use of these immunosuppressants and worsened oncologic outcomes [12]. With earlier use of tocilizumab and glucocorticoids, overall survival rates improved, and high-grade CRS decreased from approximately 9% to 4% [1]. For cases of severe CRS that do not respond to anti-IL-6 therapy or high-dose corticosteroids, anakinra is typically the preferred next option [12,17,56]. Siltuximab, a high-affinity IL-6 inhibitor, has been suggested for managing treatment-resistant CRS and may offer specific benefits for patients experiencing severe neurotoxicity. Despite its potential, it is not currently FDA-approved for CAR-T-related toxicities, and the specific impact it may have on CV outcomes remains largely unknown [56]. ICANS is a central nervous system toxicity syndrome that frequently occurs concurrently with or shortly after CRS, which explains the temporal association between neurotoxicity and CV events [17]. The American Society of Clinical Oncology (ASCO) guideline explicitly states that, in this scenario, “tocilizumab does not resolve ICANS and may worsen it,” and therefore, when CRS and ICANS occur simultaneously, management of ICANS may take precedence over management of low-grade CRS [17]. For ICANS management, ASCO guidelines position corticosteroids as the mainstay of treatment, limiting tocilizumab’s role to cases when ICANS occurs concurrently with CRS [17]. In cases refractory to corticosteroids, anakinra is the preferred next-line agent [17,58]. While clinical experience remains limited, other potential interventions include agents like ruxolitinib, cyclophosphamide, and Intravenous Immunoglobulin (IVIG), as well as more intensive measures, such as anti-thymocyte globulin (ATG), dasatinib, intrathecal chemotherapy, or the use of continuous renal replacement therapy (CRRT) for extracorporeal cytokine adsorption.

### 6.3. Post-Treatment Evaluation

**Figure 3:**
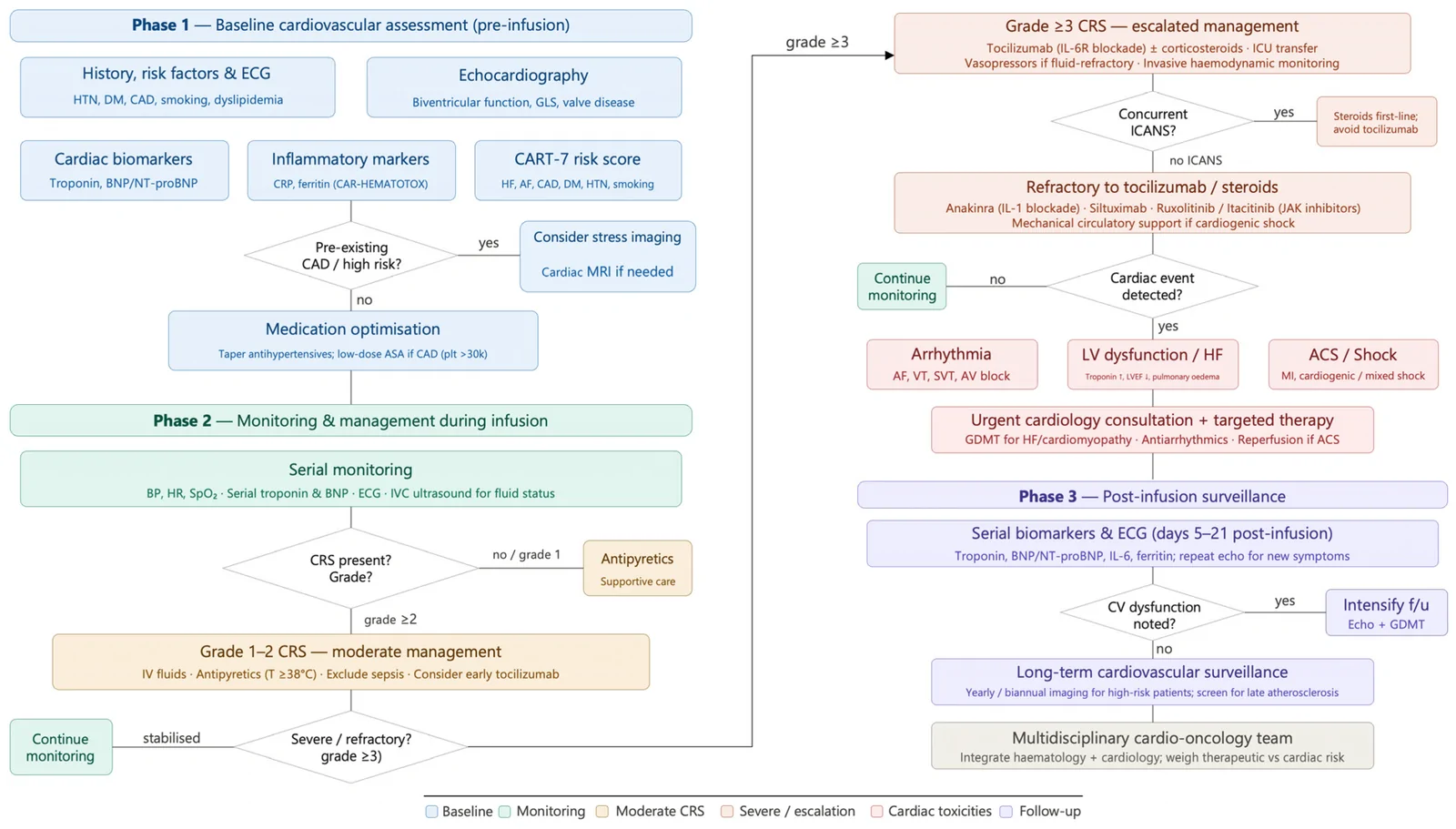
Clinical management flowchart. The flowchart depicts the continuum of cardiovascular care across three phases: pre-treatment baseline assessment and risk stratification, including echocardiographic evaluation, cardiac biomarker testing, and cardiovascular optimization; intra-treatment monitoring with prompt recognition and management of cytokine release syndrome and hemodynamic instability; and post-treatment surveillance with serial imaging and biomarker follow-up in high-risk patients. AF, atrial fibrillation; ACS, acute coronary syndrome; ASA, aspirin; CAD, coronary artery disease; CRS, cytokine release syndrome; GDMT, guideline-directed medical therapy; HF, heart failure; HTN, hypertension; ICANS, immune effector cell-associated neurotoxicity syndrome; IVC, inferior vena cava; LVEF, left ventricular ejection fraction; MI, myocardial infarction.

Post-infusion monitoring for CAR-T patients should include serial ECGs, diagnostic imaging like repeat echocardiography for new symptoms, and the frequent tracking of cardiac biomarkers such as troponins and natriuretic peptides to identify myocardial stress [41,59]. Monitoring inflammatory markers like IL-6, IL-1, and ferritin can also serve as early warning signs, as elevations in these markers—alongside troponin—have been linked to an increased risk of CV complications [12,47,49]. Recent prospective data specifically highlights a correlation between elevated high-sensitivity troponin and higher all-cause mortality, suggesting that patients with these laboratory findings require more aggressive surveillance and early therapeutic intervention [60]. For patients who developed CV complications during infusions, Baldassarre and colleagues’ group suggests that yearly or biannual imaging follow-up after completion of therapy is reasonable, especially in high-risk patients (those with CV risk factors, high-grade CRS, or documented cardiac dysfunction) [52]. The interval can be decreased if cardiac dysfunction is noted or increased in low-risk patients in whom cardiac function remains stable [52]. The flowchart for clinical management is shown in Figure 3.

## 7. Gaps in Knowledge and Future Perspectives

### 7.1. Long-Term Toxicities, Cardiovascular Monitoring and Stratification

Long-term CV toxicities after CAR-T therapy remain incompletely characterized, but emerging data suggest that cardiac dysfunction can persist in up to 50% of patients who develop cardiomyopathy [16]. Potential late cardiovascular concerns include whether altered immune function and persistent CAR-T cell circulation could lead to accelerated atherosclerosis, metabolic syndrome, hypertension, arrhythmias, persistent left ventricular dysfunction or late-onset cardiomyopathy—questions that can only be answered through longitudinal studies [12,16].

A recent literature review noted that long-term effects beyond one year post-treatment remain largely unknown, highlighting a critical knowledge gap [33]. Moreover, a review published in *Nature* in 2026 notes that as the variety of T-cell therapies and indications expand, a more nuanced understanding of potential CV toxicities and their long-term sequelae is required [18]. The lack of validated cardio-oncology risk models specific to CAR-T therapy represents another gap, though tools like the CART-7 risk score for baseline prediction and troponin monitoring for acute risk stratification show promise [47,55]. Long-term toxicity data for BiTEs are even more limited than for CAR-T, as these agents are newer and administered continuously or intermittently rather than as a one-time infusion. There are no standardized guidelines for baseline CV assessment before CAR-T or BiTE therapy [52]. While NCCN (National Comprehensive Cancer Network) recommends baseline echocardiography for CAR-T and cardiology consultation if prior cardiac history exists, the use of techniques like Global Longitudinal Strain is well established. The optimal pre-treatment workup—including the role of stress testing in high-risk patients, baseline biomarkers (troponin, BNP), and advanced imaging—remains undefined [16,52]. Biomarker-guided monitoring is particularly understudied. Troponin elevation is associated with subsequent CV events in CAR-T recipients, but prospective validation of troponin surveillance protocols is lacking. The optimal frequency and duration of cardiac biomarker monitoring post-therapy is unknown [16,52]. Long-term CV surveillance protocols are absent. Current recommendations suggest “yearly or biannual imaging follow-up” for high-risk patients after cardiotoxic therapies, but this is extrapolated from other cancer treatments rather than CAR-T/BiTE-specific data [52]. The duration of elevated CV risk beyond the acute period (30 days for CAR-T, during active treatment for BiTEs) is unknown [12,33].

### 7.2. Clinical Trial Design Gaps

Prospective CV endpoints are rarely included in CAR-T or BiTE trials. To standardize and optimize the monitoring and management of CV toxicity, the use of biomarker and CV imaging should be validated with prospective studies in terms of timing and frequency. Studies evaluating cardioprotective strategies (prophylactic beta-blockers, statins, venous thromboembolism [VTE] prophylaxis), optimal CRS management timing to prevent cardiac events, and the role of early tocilizumab are needed but largely absent [16]. To date, no randomized trials have compared prophylactic tocilizumab, anakinra, or corticosteroids against standard management for preventing CV events [1]. Standardized toxicity grading and reporting for CV events in immunotherapy trials is lacking, making cross-study comparisons difficult [61].

## 8. Conclusions

The integration of CAR-T cell therapy and BiTEs into hematologic oncology marks a definitive shift toward a “proactive cardio-protection” model. Clinical strategies should prioritize a multi-layered risk stratification framework, transitioning from simple clinical observation to the formal validation and implementation of tools like the CART-7 score alongside novel biomarkers. A primary strategic priority involves the proactive management of pre-existing CV comorbidities and risk factors—such as hypertension, dyslipidemia, diabetes, and CAD—through the optimized use of currently available, evidence-based cardiovascular therapies, with the goal of improving cardiovascular reserve before and during T-cell-redirecting immunotherapy.

The future of CV management in CAR-T and BiTEs is shifting from reactive toxicity management to proactive, personalized cardio-protection [12,18,62], driven by integrated cardio-oncology care, biomarker surveillance, and AI/machine learning for risk prediction, as highlighted by the AHA 2025 Scientific Statement [63]. A key innovation involves modifying the CAR-T construct itself (e.g., altering antigen-binding affinity) to reduce CRS and ICANS without losing efficacy [1], while the pharmacological pipeline for CRS-associated cardiotoxicity is expanding beyond tocilizumab and corticosteroids to include targeted immunomodulators like anakinra (IL-1 blockade), siltuximab (IL-6 neutralization), emapalumab (IFN-γ target), and JAK inhibitors (ruxolitinib, itacitinib), which block multiple cytokine pathways to prevent myocardial depression and capillary leak [1,64]. Several critical questions regarding CV management in the context of CAR-T and BiTE therapies remain unanswered [16], including the optimal timing and duration of guideline-directed medical therapy after CRS cardiomyopathy and the prophylactic efficacy of beta-blockers, ACE inhibitors, or emerging drugs like Sodium–Glucose Cotransporter 2 inhibitors and Glucagon-Like Peptide-1 Receptor Agonists [65,66], which can effectively reduce adverse CV events. Furthermore, the true incidence of CRS-independent CV toxicity—which accounted for 31.7% of CV adverse events in FAERS data—remains unclear, as do the most effective pharmacologic and mechanical VTE prevention strategies during and after treatment. Finally, research is needed to establish whether prior exposure to ICIs serves as a compounding risk factor for CV complications in patients subsequently receiving CAR-T or BiTE therapies [16]. The next generation of therapeutic design should address the “off-target, off-tumor” mechanisms—such as titin cross-reactivity—through the adoption of adaptor CAR-T (AdCAR-T) systems and BiTE prodrugs that are selectively activated within the tumor microenvironment. These bioengineering innovations offer a critical avenue to decouple antitumor potency from systemic inflammatory collateral damage. As patient survival extends, the clinical mandate must expand to include standardized longitudinal surveillance focused on the emerging threat of accelerated atherosclerosis and chronic vascular inflammation. Ultimately, establishing an integrated, multidisciplinary framework is essential to ensuring that the curative potential of immune redirection is not undermined by preventable, late-onset CV morbidity. In summary, the field is moving toward a precision cardio-oncology model that integrates AI-driven risk prediction, novel biomarkers, advanced imaging, engineering of safer CAR constructs, and expanded cytokine-targeted pharmacotherapy—but prospective, randomized data remain critically needed to validate these approaches.

## Figures and Tables

**Table 3:** Comparative overview of cardiovascular toxicity between CAR-T and BiTE therapies.

Parameter	CAR-T	BiTEs
**Major therapeutic targets and representative products**	CD19 (e.g., tisagenlecleucel, axicabtagene ciloleucel) and BCMA (e.g., idecabtagene vicleucel) are the most established targets; additional targets under development (CD20, CD22, CD138)	CD19 × CD3 (blinatumomab), CD20 × CD3 (epcoritamab, glofitamab, mosunetuzumab), BCMA × CD3 (teclistamab, elranatamab), GPRC5D × CD3 (talquetamab)
**Patterns of T-cell activation**	Activation via an engineered chimeric receptor produced ex vivo, MHC-independent; single infusion with prolonged cellular expansion and persistence	Polyclonal activation via simultaneous CD3/tumor antigen binding (bridging), MHC-independent; requires continuous/intermittent administration (“off-the-shelf” agents)
**Relationship with CRS**	Dominant mechanism of cardiovascular toxicity: most CV events are closely associated with grade ≥ 2 CRS	More complex relationship: a substantial proportion (up to ~83% in some FAERS datasets) of CV events occur independently of CRS, suggesting CRS-independent mechanisms not yet well characterized
**Major cardiovascular toxicities**	Hypotension, tachycardia, supraventricular/ventricular arrhythmias, left ventricular dysfunction, cardiomyopathy, heart failure, cardiac arrest, rare myocardial infarction	Hypotension, tachycardia, atrial fibrillation/flutter, left ventricular dysfunction, shock, myocarditis (disproportionate signal with teclistamab), pericarditis, thromboembolic events, rare sudden death
**Typical timing of onset**	Early, typically 5–21 days post-infusion; CRS usually at 2–3 days (ranging from hours to 15 days)	Early, median 5–21 days; in multiple myeloma, mainly within the first month
**Risk factors**	High-grade CRS, delayed CRS treatment, pre-existing CV comorbidities, elevated IL-6/troponin/ferritin/CRP levels	Older age, male sex, weight loss, CRS, respiratory failure, pre-existing CAD
**Monitoring parameters**	Serial ECGs, echocardiography, troponin, BNP/NT-proBNP, IL-6, ferritin, CRP; dedicated scores such as CART-7 and CAR-HEMATOTOX	Similar (ECG, cardiac biomarkers, imaging), but protocols are less standardized; long-term surveillance data even more limited than for CAR-T
**Management strategies**	Fluid therapy, vasopressors if needed, tocilizumab (first line for CRS), corticosteroids (second line), anakinra/siltuximab in refractory cases; differentiated management if ICANS coexists	Similar approach based on CRS management (tocilizumab, corticosteroids), but less specific evidence for the CRS-independent component of CV toxicity
**Strength of supporting evidence**	Stronger: numerous pivotal trials (ZUMA-1, JULIET, KarMMa) and dedicated meta-analyses/registries (e.g., Koeckerling 2024 [46], Maleki 2024 [42])	Weaker/more heterogeneous: evidence mainly derives from pharmacovigilance data (FAERS) and a more limited number of dedicated observational studies, with potential under/overreporting bias

Abbreviations: BCMA, B-cell maturation antigen; BiTEs, bispecific T-cell engagers; BNP, B-type Natriuretic Peptide; CAD, coronary artery disease; CAR-T, chimeric antigen receptor T-cell; CRP, C-reactive protein; CRS, cytokine release syndrome; CV, cardiovascular; FAERS, FDA Adverse Event Reporting System; GPRC5D, G Protein-Coupled Receptor Class C Group 5 Member D; ICANS, immune effector cell-associated neurotoxicity syndrome; IL-6, Interleukin-6; NT-proBNP, N-terminal pro-B-type Natriuretic Peptide.

## Data Availability

No new data were created or analyzed in this study. Data sharing is not applicable to this article.

## Abbreviations

The following abbreviations are used in this manuscript:

ACE	Angiotensin-Converting Enzyme
ACS	Acute Coronary Syndrome
AdCAR-T	Adaptor Chimeric Antigen Receptor T-cell
Adj.ROR	Adjusted Reporting Odds Ratio
AE	Adverse Event
AF	Atrial Fibrillation
AHA	American Heart Association
AI	Artificial Intelligence
ALL	Acute Lymphoblastic Leukemia
ASA	Aspirin
ASCO	American Society of Clinical Oncology
ATG	Anti-Thymocyte Globulin
BCMA	B-cell Maturation Antigen
BiTEs	Bispecific T-cell Engagers
BNP	B-type Natriuretic Peptide
CAD	Coronary Artery Disease
CAR-T	Chimeric Antigen Receptor T-cell
Cas9	CRISPR-associated protein 9
CD20	Cluster of Differentiation 20
CD22	Cluster of Differentiation 22
CI	Confidence Interval
CRISPR	Clustered Regularly Interspaced Short Palindromic Repeats
CRP	C-Reactive Protein
CRRT	Continuous Renal Replacement Therapy
CRS	Cytokine Release Syndrome
CV	Cardiovascular
CVAEs	Cardiovascular Adverse Events
CVD	Cardiovascular Disease
DIC	Disseminated Intravascular Coagulation
DLBCL	Diffuse Large B-cell Lymphoma
ECG	Electrocardiogram
EF	Ejection Fraction
EMA	European Medicines Agency
FAERS	FDA Adverse Event Reporting System
FDA	Food and Drug Administration
GDMT	Guideline-Directed Medical Therapy
HF	Heart Failure
HR	Hazard Ratio
HTN	Hypertension
ICAHT	Immune Effector Cell-Associated Hematotoxicity
ICANS	Immune Effector Cell-Associated Neurotoxicity Syndrome
ICIs	Immune Checkpoint Inhibitors
IFN-γ	Interferon gamma
IL-1β	Interleukin-1 beta
IL-6	Interleukin-6
IL-8	Interleukin-8
IVIG	Intravenous Immunoglobulin
JAK	Janus Kinase
LV	Left Ventricular
LVEF	Left Ventricular Ejection Fraction
MACEs	Major Adverse Cardiovascular Events
MI	Myocardial Infarction
MRD	Minimal Residual Disease
NCCN	National Comprehensive Cancer Network
NT-proBNP	N-terminal pro-B-type Natriuretic Peptide
OR	Odds Ratio
PD-1	Programmed Cell Death protein 1
PD-L1	Programmed Cell Death Ligand 1
PMBCL	Primary mediastinal Large B-cell Lymphoma
RCT	Randomized Controlled Trial
TFL	Transformed Follicular Lymphoma
TNF-α	Tumor Necrosis Factor-alpha
VTE	Venous Thromboembolism
